# Evaluation of high levels of solvent extracted *Camelina sativa* meal in diets on performance, blood indices, cecal microorganisms, and nutrient digestibility in broilers

**DOI:** 10.1007/s11250-025-04339-1

**Published:** 2025-03-06

**Authors:** Sakine Yalçın, Muhammad Shazaib Ramay, Hüseyin Yalçınkaya, Özlem Kardoğan, Ali Erkurt, Bahadır Kılınç, Emre Sunay Gebeş, Atakan Bundur, Esin Ebru Onbaşılar, Suzan Yalçın, İlhan Subaşı, Celalettin Etkin Şafak, Elif Kocadaş

**Affiliations:** 1 Department of Animal Nutrition and Nutritional Diseases, Faculty of Veterinary Medicine, Ankara University, Ankara, Turkey; 2 Department of Border Control for Animal and Animal Products, Directorate General for Food and Control, Ministry of Agriculture and Forestry, Ankara, Turkey; 3 Poultry Disease Diagnosis Laboratory, Veterinary Control Central Research Institute, Ankara, Turkey; 4 Biochemistry Laboratory, Veterinary Control Central Research Institute, Ankara, Turkey; 5 Pathology Laboratory, Veterinary Control Central Research Institute, Ankara, Turkey; 6 Graduate School of Health Sciences, Ankara University, Ankara, Turkey; 7 Department of Animal Husbandry, Faculty of Veterinary Medicine, Ankara University, Ankara, Turkey; 8 Department of Food Hygiene and Technology, Faculty of Veterinary Medicine, Selçuk University, Konya, Turkey; 9 Department of Seed Science and Technology, Faculty of Agriculture, Bolu Abant İzzet Baysal University, Bolu, Turkey; 10 Department of Physiology, Faculty of Veterinary Medicine, Ankara University, Ankara, Turkey; 11 Eşme District Directorate of Agriculture and Forestry, Uşak, Turkey

**Keywords:** Antinutrients, By-products, Digestibility, Oilseed, Performance

## Abstract

This study aimed to investigate the influence of utilizing high levels of solvent extracted *Camelina sativa* meal (*Camelina sativa* (L.) Crantz, Arslanbey cultivar) in broilers. A total of 270 Ross 308 male chicks were randomly distributed into five groups. Solvent extracted *Camelina sativa* meal was used at the levels of 0, 10, 15, 20 and 25%. The trial lasted 42 days. Significant linear reduction was observed in live weight gain, feed consumption, feed efficiency, European Production Efficiency Factor, and carcass yield. The relative weight percentages of the heart, proventriculus, gizzard, and thyroid gland, as well as the heterophils/lymphocyte ratio, serum total cholesterol, albumin, IgA, and IgG levels increased linearly with the use of *Camelina sativa* meal in the diets. No significant changes were observed in free triiodothyronine (fT3) and free thyroxine (fT4) hormone levels and fT3/fT4 ratio in serum and the counts of *E. coli* and *Lactobacillus* in the cecum. Dietary *Camelina sativa* meal causes a significant reduction in villus height of the duodenum, jejunum, and ileum. It decreases the villus height/crypt depth ratio in jejunum and ileum and also the digestibility values of dry matter, crude protein, and energy. It is concluded that high levels (10, 15, 20, and 25%) of solvent extracted *Camelina sativa* meal were not suitable feedstuffs for broiler production.

## Introduction

*Camelina sativa* (L.) Crantz (false flax, gold-of-pleasure) is a promising oilseed plant having industrial potential due to the usage in the production of biopolymers, biofuels, cosmetics, agrochemicals, food, and feed (Singh et al. 2023). Recently, camelina has gained importance in terms of increasing its seed weight and seed yield, reducing anti-nutritional factors, and improving lipid quality (Zanetti et al. 2021). Unfortunately, camelina expellers had 70.3 g/kg soluble non-starch polysaccharides, 1.60 mg/g tannins, 32.8 g/kg erusic acid (as a proportion of total oil), 500 mg/kg allyl-isothiocyanate, and 4.24 units/g trypsin inhibitor (Cerisuelo et al. 2023). Meza et al. (2022) determined the concentration of average total glucosinolates (GSLs) to be 8.39 mg/g in camelina seeds and 15.95 mg/g in the defatted camelina meal. Pagliari et al. (2022) reported the concentration of total GSLs as 5.69 mg/g in camelina-defatted meals. The range of phytic acid content was between 21.0 and 24.8 g/kg and sinapine content was found to be 2.19–3.21 g/kg in ten solvent extracted *Camelina sativa* meal samples (Colombini et al. 2014). The most important antinutritive compounds in camelina meal are GSLs, which are unpalatable and affect especially thyroid function (Tripathi and Mishra 2007). High levels of erusic acid may cause fatty acid degeneration of the heart, adrenal gland, kidney and thyroid (Budin et al. 1995). *Camelina sativa* meal is high in protein. Colombini et al. (2014) evaluated ten solvent extracted *Camelina sativa* meal samples and found that the meal contained 42.0–48.4% crude protein (CP), 4.17–9.33% ether extract (EE) and 5.85–6.67% ash on a dry matter (DM) basis. However, the nutrient profiles of *Camelina sativa* meal can vary due to cultivar, season, and other agronomic factors such as soil type and processing method (Cherian 2012).

*Camelina sativa* has previously been used in the broiler diets (Ryhanen et al. 2007; Pekel et al. 2009, 2015; Aziza et al. 2010; Thacker and Widyaratne 2012). In these studies, *Camelina sativa* meal/cake used in the diets was produced after pressing without solvent extraction and therefore it contains oil higher than 6%. The content of antinutritional factors might be affected by production techniques. Pekel et al. (2015) determined that the effects of 10% and 20% camelina meal have the effect of about 13% EE for 3-week-old broilers, that high viscosity in jejunal digesta was demonstrated and that utilization of energy and nitrogen in camelina meal by broilers is low; that may be due to the total GSLs content. Oryschak et al. (2020) stated that dietary inclusion (up to 24%) of *Camelina sativa* cake having 34% CP and 22% EE is a safe feed ingredient for broilers, with no negative impact on their health. However, there is a lack of information on the use of high levels of solvent extracted *Camelina sativa* meal (CSM) containing less than 3% oil in broilers. In Turkey, *Camelina sativa* seeds are used for biodiesel production, and solvent extraction technique by hexane is used to extract oil. The residual meal contained oil less than 3%. We hypothesized that solvent extracted CSM could partially replace soybean meal/fullfat soya as a protein supplement in broiler diets at the inclusion levels of 10, 15, 20 or 25%. Therefore the effects of the usage of high levels of solvent extracted CSM on growth performance, blood indices, cecal microorganisms, intestinal histomorphology, and nutrient digestibility in broilers were examined in the present study.

## Materials and methods

All animal care and use procedures were approved by the Animal Ethics Committee of Ankara University, Turkey (2019–6–62).

### Experimental design and diets

A total of 270 Ross 308 male broiler chicks were randomly assigned to five groups, with each group comprising 54 chicks. Chicks were spray-vaccinated in the hatchery before arrival. The formulations of diets were designed according to the Ross 308 breeding catalogue (Aviagen Inc. 2014). Accordingly, a starter diet of 23% CP and 3000 kcal/kg metabolizable energy (ME) for 0–10 days of age, a grower diet of 21.5% CP and 3100 kcal/kg ME for 10–24 days of age, and a finisher diet of 19.5% CP and 3200 kcal/kg ME for 24–42 days of age were prepared as presented in Table 1. The control group diet was prepared based on maize and soybean meal/fullfat soya. The diets were formulated to contain 0, 10, 15, 20, and 25% *Camelina sativa* meal. CSM was obtained from the oil extraction of *Camelina sativa* (*Camelina sativa* (L.) Crantz, Arslanbey cultivar registered by Field Crops Central Research Institute in Turkey) for biodiesel. In the first stage of oil extraction, pre-cleaning of *Camelina sativa* seeds was done, and then the seeds were crushed by passing through the rollers into flakes. Afterwards, the flakes were transferred to the pan having a temperature of 80–90 °C at the top of the 5-layered pans. Water vapour was sprayed on the flakes and the moisture content was increased to 16–18%. The flakes come from the top pan to the bottom pan by roasting in 20–30 min. This material from the bottom pan, which is at a temperature of 110–115 °C and 4–4.5% moisture, is poured into the press. The amount of oil remaining was about 8–10% in the cake after being squeezed in presses. Then it was sent to the extractors and extracted by using hexane. The amount of oil in the obtained extracted meal was less than 3%.

**Table 1:** Ingredients and chemical composition of the diets^a^ (as-fed basis)

Ingredients (%)	Starter (0–10 days)					Grower (10–24 days)					Finisher (24–42 days)				
	Solvent extracted CSM (%)					Solvent extracted CSM (%)					Solvent extracted CSM (%)				
	0	10	15	20	25	0	10	15	20	25	0	10	15	20	25
Maize	52.62	48.42	46.18	44.03	41.93	55.09	50.57	48.50	46.49	44.53	58.93	54.80	52.81	50.85	48.82
Fullfat soya (37% CP)	13.14	21.12	24.90	26.71	26.40	19.78	26.51	26.77	25.12	22.51	20.36	19.96	17.52	15.19	13.67
Soybean meal (47% CP)	29.48	15.70	9.04	3.93	0.51	20.53	7.92	4.03	1.64	0.00	15.41	8.36	6.59	4.73	2.24
CSM (37.88% CP)	0.00	10.00	15.00	20.00	25.00	0.00	10.00	15.00	20.00	25.00	0.00	10.00	15.00	20.00	25.00
Soya oil	0.00	0.00	0.12	0.57	1.40	0.00	0.40	1.10	2.15	3.36	1.00	2.58	3.78	4.93	5.97
Monocalcium phosphate	2.00	2.00	2.00	2.00	2.00	2.00	2.00	2.00	2.00	2.00	1.90	1.90	1.90	1.90	1.90
Limestone	1.50	1.50	1.50	1.50	1.50	1.40	1.40	1.40	1.40	1.40	1.30	1.30	1.30	1.30	1.30
Salt	0.25	0.25	0.25	0.25	0.25	0.25	0.25	0.25	0.25	0.25	0.25	0.25	0.25	0.25	0.25
Sodium bicarbonate	0.10	0.10	0.10	0.10	0.10	0.10	0.10	0.10	0.10	0.10	0.10	0.10	0.10	0.10	0.10
DL-Methionine	0.30	0.30	0.30	0.30	0.30	0.30	0.30	0.30	0.30	0.30	0.25	0.25	0.25	0.25	0.25
L-Lysine	0.20	0.20	0.20	0.20	0.20	0.20	0.20	0.20	0.20	0.20	0.15	0.15	0.15	0.15	0.15
Threonine	0.10	0.10	0.10	0.10	0.10	0.10	0.10	0.10	0.10	0.10	0.10	0.10	0.10	0.10	0.10
Vitamin premix^b^	0.15	0.15	0.15	0.15	0.15	0.15	0.15	0.15	0.15	0.15	0.15	0.15	0.15	0.15	0.15
Mineral premix^c^	0.10	0.10	0.10	0.10	0.10	0.10	0.1	0.10	0.10	0.10	0.10	0.10	0.10	0.10	0.10
Anticoccidial^d^	0.06	0.06	0.06	0.06	0.06										
Chemical composition (analyzed values)															
Dry matter (%)	90.82	91.07	90.10	91.34	90.40	90.86	91.09	91.12	90.06	90.45	90.81	91.21	91.29	90.49	90.72
Crude protein (%)	23.08	23.12	23.10	22.96	23.03	21.49	21.55	21.56	21.51	21.48	19.42	19.48	19.44	19.43	19.45
Ether extract (%)	5.06	6.33	7.42	8.17	8.80	6.42	8.10	8.85	9.45	9.98	7.57	8.88	9.39	10.19	10.71
Crude fibre (%)	3.66	4.27	4.57	4.90	5.12	3.55	4.14	4.36	4.67	4.89	3.32	3,93	4,06	4.32	4.60
Crude ash (%)	6.39	6.41	6.43	6.57	6.68	6.10	6.16	6.30	6.38	6.42	5.77	5.88	5.99	6.08	6.10
Calcium (%)	1.22	1.23	1.23	1.23	1.24	1.18	1.19	1.19	1.19	1.20	1.07	1.08	1.09	1.08	1.09
Phosphorus (%)	0.74	0.75	0.76	0.76	0.76	0.68	0.68	0.68	0.69	0.69	0.65	0.66	0.66	0.66	0.67
ME^e^ (kcal/kg)	3006	2998	3000	3000	3004	3110	3102	3106	3102	3105	3209	3204	3202	3202	3200
Viscosity (cP)	0.28	0.35	0.38	0.39	0.42	0.31	0.33	0.34	0.36	0.39	0.33	0.39	0.42	0.44	0.47

*CSM* *Camelina sativa* meal, *ME *metabolizable energy

^a^1% celite was added to each mixed feed to determine digestibility of nutrients

^b^ 1.5 kg vitamin premix contains 11.000.000 IU vitamin A, 3.500.000 IU vitamin D_3_, 100 g vitamin E, 3 g vitamin K_3_, 3 g vitamin B_1_, 6 g vitamin B_2_, 15 g calcium D pantothenate, 1 g vitamin B_6_, 35 g niacin, 1.5 g folic acid, 200 mg biotin ve 20 mg vitamin B_12_

^c^1 kg mineral premix contains 30 g copper, 120 g manganese, 110 g zinc, 2 g iodine, 300 mg selenium, 50 g iron

^d^Salinomycin

^e^Calculated

Each group had six subgroups containing 9 chicks each. Thus, a trial was conducted in a total of 30 pens (90 × 80 cm). Wood shavings were placed in each pen as litter. Feed and water were provided for ad libitum consumption and the diets were presented in mash form. The trial lasted 42 days. A 23-h light and 1-h dark period was applied in the first week of the trial. After the first week, the lighting will be gradually reduced within five days to an 18-h light and 6-h dark program.

### Analysis of *Camelina sativa* meal and diets

The nutrient compositions of solvent extracted CSM and diets were determined according to the AOAC (2000). Metabolizable energy values were estimated based on the methodology described by Yalçın et al. (2008). Calcium and phosphorus levels were determined (King and Sheridan 2019) with ICP-MS (Agilent 7700 × ICP-MS). Total GSLs (Mawlong et al. 2017), and sinapin (Cai and Arntfield 2001) levels of CSM were analyzed.

### Growth performance

The live weights of the birds were determined by weighing them individually at the beginning of the study (day 0), at the times when the feed was changed (days 10, 24), and at the end of the trial (day 42). Weight gain was determined by calculating the differences between initial and final weights. Feed intake was measured on a subgroup basis, and the feed conversion ratio (FCR) was expressed as the amount of feed consumed per kilogram of weight gained. Daily monitoring of the birds was conducted, and livability, along with European Production Efficiency Factor (EPEF) values, was computed following the method outlined by Onbaşılar et al. (2023).

### Slaughtering and collection of samples

On the 24th day of the experiment, six broilers from each group were slaughtered by severing the jugular vein for histomorphological evaluation. Duodenum, jejunum, and ileum segments were removed from their mid points, flushed with physiological saline solution, and submerged in 10% formalin solution.

On the 42nd day of the experiment, 12 broilers from each group were weighed, and blood samples were collected from the wing vein into two vacuum tubes (with EDTA and without anticoagulant). They were slaughtered by severing the jugular vein. Hot carcasses, liver, proventriculus, gizzard, spleen, heart, thyroid gland, and abdominal fat were weighed to determine the carcass yield and relative weights of internal organs. The digesta contents of the jejunum and ileum were collected into tubes to determine viscosity. The cecal content of the birds was taken into sterile tubes for molecular analysis of bacterial community structure using HRM and QPCR methods, They were stored at −80 °C until analyzed.

### Determination of nutrient digestibility and analysis

The digestive ratios of DM, gross energy, and CP were determined using the external inert marker according to the method of Wetherbee and Gruber (1993). Celite as an inert marker was added at the level of 1% to the diets. Clean excreta (free from feathers, feed, and litter) were collected on days 9 and 10, 24 and 25, and 41 and 42 by placing plastic material above litter in each pen for one hour each time. Samples collected 4 times (2 in the morning and 2 in the afternoon) were stored at −18 °C. Analyses were determined as reported by Aziza et al. (2013).

### Viscosity analysis

Jejunal and ileal digesta samples were homogenized and centrifuged at 5.000 × g for 20 min at 4 °C. Then supernatants were taken into eppendorf tubes and centrifuged at 12.000 × g for 10 min at 4 °C. A 0.5 ml sample of the supernatant was used to measure viscosity using a viscometer (model DV-II + Pro, Brookfield Digital Viscometer, Brookfield Engineering Laboratories Inc., Stoughton, MA, USA) in centipoise (cP) as stated by Yalçın et al. (2017).

### Histological analysis

Intestinal segments were processed for morphological analysis following the methods described by Onbaşılar et al. (2024). Measurements of villus height (VH, from the tip of the villi to the villus-crypt junction), villus width (at the mid-point of the villi), and crypt depth (CD, the depth of the invagination between adjacent villi) were performed using a Leica DM2500 microscope with Leica Application Suite V4.12.0 software. Ten well-oriented villus-crypt units per intestinal sample were randomly selected and measured. The villus height to crypt depth ratio (VH/CD) was then calculated for each sample.

### Microbiological analysis

The cecal samples were pretreated to remove dead cell DNA present in the samples, thus only live bacterial species were included in the analysis (Villarreal et al. 2013). Then, total DNA was isolated from the samples. HRM analysis was applied to the isolated DNA to reveal the bacterial community profiles of the samples. qPCR was applied to the isolated DNA to determine the total bacteria, *Lactobacillus*, *Enterococcus*, and *E. coli* counts present in the samples (Livak and Schmittgen 2001).

### Analysis of blood samples

Blood in the tubes containing EDTA was collected to determine some haematological parameters after sampling. The microhaematocrit technique was used for haematocrit (HTC) determination (Gebretsadkan et al. 2015) and hemoglobin (Hb) was measured by the Sahli method (Patil et al. 2013). The leukogram technique was performed to express the percentages of heterophiles (H), lymphocytes (L), eosinophiles, basophiles, and monocytes (Panhwer et al. 2016). Then the ratio of H/L was calculated. Blood samples without anticoagulant were centrifuged at 3220xg for 8 min and serum was removed for analysis of total protein (Otto Scientific, OttoBC154), albumin (Otto Scientific, OttoBC123) total cholesterol (Otto Scientific, OttoBC135), triglycerides (Otto Scientific, OttoBC155), total antioxidant status (TAS, Rel Assay, RL0017) and total oxidant status (TOS, Rel Assay, RL0024) were determined with an autoanalyzer (Mindray-BS400). Oxidative stress index (OSI) was calculated as described by Ramay and Yalçın (2020). Free triiodothyronine (fT3, Elabscience, E-EL-0079) and free thyroxine (fT4, Elabscience, E-EL-0122) levels were determined with ELISA (microplate reader: BIO-TEK ELx800-Aotu strip washer: BIO TEK ELx50). The ratio of fT3/fT4 was calculated. Immunoglobulin G levels (IgG, BT-LAB, E0019Ch) were determined with ELISA (microplate reader: BIO-TEK ELx800-Aotu strip washer: BIO TEK ELx50). Levels of Immunoglobulin A (IgA, Otto Scientific, OttoBC146) and Immunoglobulin M (IgM, Otto Scientific, OttoBC149) were determined with an autoanalyzer (Mindray-BS400).

### Statistical analysis

Statistical analyses were conducted using SPSS software (SPSS Inc., Chicago, IL, USA). Data distribution was assessed for normality with the Kolmogorov–Smirnov test. To evaluate the impact of varying CSM levels on different parameters, one-way ANOVA was employed. Mean differences among groups were examined using Tukey’s test. Linear and quadratic effects were analyzed using polynomial contrasts. Data on microorganisms were first converted to logarithm and then analyzed as in other data above. The level of significance was taken as *p* < 0.05 (Dawson and Trapp 2001).

## Results

As a result of the analysis made, it was found that *Camelina sativa* (Arslanbey cultivar) meal had 90.95% DM, 37.88% CP, 2.61% EE, 10.80% crude fibre, and 6.34% crude ash. CSM had 26.84 µmoles/g total GSLs and 2036 mg/kg sinapin.

A statistically significant linear reduction (*p* < 0.001) was observed in live weight, live weight gain, and feed consumption in broilers starting from day 10 with the use of different levels of solvent extracted camelina meal in diets as seen in Table 2. The values of live weight and live weight gain in the groups fed solvent extracted CSM were lower than those of control group after 24th day of the experiment (*p* < 0.001). The CSM groups consumed less feed than the control group during the experiment (*p* < 0.001). The use of 10, 15, 20, and 25% solvent extracted camelina meal in the diets negatively (*p* < 0.001) affected feed utilization and EPEF. There was one death in each group containing 15 and 25% camelina meal during the experiment, and pathological examination revealed that the deaths were not related to feed.

**Table 2:** Effects of high levels of solvent extracted CSM on performance parameters in broilers

Day	Solvent extracted CSM (%)					*p* value		
	0	10	15	20	25	Combined	Linear	Quadratic
Live weight (g)								
0	42.93 ± 0.15	42.92 ± 0.13	42.82 ± 0.15	42.97 ± 0.16	42.90 ± 0.12	0.958	0.984	0.788
10	230.69 ± 3.25^a^	221.92 ± 2.05^ab^	223.40 ± 2.44^ab^	216.65 ± 2.78^b^	196.71 ± 2.68^c^	< 0.001	< 0.001	0.005
24	970.14 ± 13.46^a^	877.55 ± 13.01^b^	839.73 ± 16.17^bc^	795.66 ± 13.31^c^	722.41 ± 14.50^d^	< 0.001	< 0.001	0.544
42	2859.18 ± 46.84^a^	2612.48 ± 46.47^b^	2468.68 ± 37.87^b^	2258.34 ± 32.48^c^	1965.55 ± 49.37^d^	< 0.001	< 0.001	0.333
Live weight gain (g/period)								
10–0	187.76 ± 3.19^a^	179.00 ± 2.00^ab^	180.58 ± 2.36^ab^	173.68 ± 2.83^b^	153.81 ± 2.66^c^	< 0.001	< 0.001	0.004
24–10	739.45 ± 10.74^a^	655.63 ± 11.67^b^	616.34 ± 15.27^bc^	579.01 ± 11.24^c^	525.71 ± 12.09^d^	< 0.001	< 0.001	0.181
24–0	927.21 ± 13.36^a^	834.63 ± 12.93^b^	796.91 ± 16.09^bc^	752.69 ± 13.28^c^	679.51 ± 14.49^d^	< 0.001	< 0.001	0.544
42–24	1889.04 ± 35.14^a^	1734.93 ± 35.94^b^	1628.95 ± 25.64^b^	1462.68 ± 23.50^c^	1243.14 ± 36.75^d^	< 0.001	< 0.001	0.120
42–0	2816.25 ± 46.74^a^	2569.56 ± 46.42^b^	2425.86 ± 37.75^b^	2215.37 ± 32.46^c^	1922.65 ± 49.37^d^	< 0.001	< 0.001	0.332
Feed consumption (g/period)								
10–0	264.49 ± 4.16^a^	251.30 ± 3.14^b^	252.24 ± 3.33^b^	247.35 ± 3.19^b^	227.21 ± 3.43^c^	< 0.001	< 0.001	0.038
24–10	1129.59 ± 14.36^a^	1066.29 ± 12.44^b^	1045.55 ± 13.84^b^	1025.40 ± 17.44^b^	961.79 ± 19.73^c^	< 0.001	< 0.001	0.243
24–0	1394.08 ± 18.01^a^	1317.60 ± 12.85^b^	1297.79 ± 14.40^b^	1272.75 ± 19.45^b^	1189.00 ± 22.76^c^	< 0.001	< 0.001	0.141
42–24	3131.19 ± 18.29^a^	3058.87 ± 19.28^b^	3055.20 ± 21.35^b^	3033.51 ± 15.09^bc^	2982.62 ± 15.81^c^	< 0.001	< 0.001	0.222
42–0	4525.26 ± 36.16^a^	4376.47 ± 26.13^b^	4352.99 ± 34.15^b^	4306.27 ± 21.38^b^	4171.63 ± 35.80^c^	< 0.001	< 0.001	0.110
Feed conversion ratio (kg feed intake/kg live weight gain)								
10–0	1.41 ± 0.01^b^	1.40 ± 0.02^b^	1.40 ± 0.01^b^	1.42 ± 0.01^b^	1.48 ± 0.01^a^	0.001	0.001	0.004
24–10	1.53 ± 0.01^e^	1.63 ± 0.03^d^	1.70 ± 0.03^c^	1.77 ± 0.01^b^	1.83 ± 0.02^a^	< 0.001	< 0.001	0.272
24–0	1.50 ± 0.01^e^	1.58 ± 0.02^d^	1.63 ± 0.02^c^	1.69 ± 0.01^b^	1.75 ± 0.01^a^	< 0.001	< 0.001	0.682
42–24	1.66 ± 0.02^d^	1.77 ± 0.03^cd^	1.88 ± 0.05^c^	2.08 ± 0.04^b^	2.41 ± 0.06^a^	< 0.001	< 0.001	0.001
42–0	1.61 ± 0.02^e^	1.71 ± 0.03^d^	1.80 ± 0.02^c^	1.95 ± 0.02^b^	2.18 ± 0.04^a^	< 0.001	< 0.001	0.003
Livability (%)	100.00 ± 0.00	100.00 ± 0.00	98.41 ± 1.59	100.00 ± 0.00	98.41 ± 1.59	0.566	0.325	0.380
EPEF (%)	423.93 ± 11.23^a^	365.82 ± 12.67^b^	322.91 ± 11.22^c^	276.88 ± 7.39^d^	212.52 ± 9.32^e^	< 0.001	< 0.001	0.607

*n* = 6 for each group, values within a row with different superscripts (a, b, c, d, e) differ significantly at *p* < 0.05

*CSM Camelina sativa* meal, *EPEF* European production efficiency factor

Effects of high levels of solvent extracted CSM on carcass yield and some relative organ weights are shown in Table 3. Significant linear reduction (*p* < 0.001) in carcass yield was observed with increasing levels of dietary CSM. The use of solvent extracted CSM at 10, 15, 20, and 25% levels in the diets did not create any statistically significant differences in the relative weight percentages of liver, spleen, bursa Fabricius, and abdominal fat. The relative weight percentage of the heart (*p* = 0.009), proventriculus (*p* < 0.001), gizzard (*p* < 0.001), and thyroid gland (*p* = 0.002) was found to increase linearly with the use of camelina meal in the diets.

**Table 3:** Effects of high levels of solvent extracted CSM on carcass yield and some relative organ weights in broilers

Item (g/100 g SW)	Solvent extracted CSM (%)					*p-value*		
	0	10	15	20	25	Combined	Linear	Quadratic
Carcass yield	62.78 ± 0.44^a^	61.81 ± 0.45^ab^	61.02 ± 0.33^b^	59.36 ± 0.43^c^	59.08 ± 0.35^c^	< 0.001	< 0.001	0.402
Liver	1.89 ± 0.04	1.76 ± 0.03	1.80 ± 0.04	1.86 ± 0.04	1.84 ± 0.03	0.118	0.063	0.076
Spleen	0.107 ± 0.008	0.113 ± 0.005	0.117 ± 0.006	0.119 ± 0.007	0.119 ± 0.007	0.635	0.147	0.533
Heart	0.427 ± 0.012^b^	0.441 ± 0.011^ab^	0.485 ± 0.017^a^	0.477 ± 0.012^ab^	0.463 ± 0.011^ab^	0.010	0.009	0.026
Bursa Fabricius	0.169 ± 0.007	0.174 ± 0.011	0.191 ± 0.012	0.182 ± 0.009	0.201 ± 0.008	0.124	0.019	0.422
Proventriculus	0.343 ± 0.012^c^	0.399 ± 0.009^b^	0.424 ± 0.014^ab^	0.439 ± 0.013^ab^	0.457 ± 0.013^a^	< 0.001	< 0.001	0.071
Gizzard	1.25 ± 0.03^b^	1.30 ± 0.03^ab^	1.36 ± 0.04^ab^	1.45 ± 0.05^a^	1.44 ± 0.06^a^	0.007	< 0.001	0.595
Abdominal fat	1.46 ± 0.08	1.29 ± 0.06	1.33 ± 0.06	1.31 ± 0.08	1.29 ± 0.10	0.469	0.186	0.400
Thyroid gland	0.0095 ± 0.0007^b^	0.0098 ± 0.0005^ab^	0.0107 ± 0.0007^ab^	0.0107 ± 0.0004^ab^	0.0122 ± 0.0007^a^	0.028	0.002	0.477

*n* = 12 for each group, values within a row with different superscripts (a, b, c) differ significantly at *p* < 0.05

*CSM Camelina sativa* meal, *SW* slaughtering weight

Effects of high levels of solvent extracted CSM on some haematological parameters and some blood serum biochemical indices were shown in Table 4. The use of high levels of solvent extracted camelina meal in diets did not cause any difference in haematocrit, haemoglobin, heterophils, eosinophils, basophils, and monocytes in the blood. As CSM increased in the diet, lymphocyte concentration in the blood decreased (*p* = 0.009) and the heterophil/lymphocyte ratio increased (*p* = 0.026). The use of camelina meal at 0, 10, 15, 20, and 25% levels in the diets did not cause any differences among the groups in TAS, TOS, OSI, total protein, and triglyceride in blood serum. Total cholesterol and albumin levels in serum were increased by CSM inclusion (*p* < 0.001). Serum IgA and IgG increased as the level of solvent extracted camelina meal in the diet increased (*p* < 0.05), while serum IgM levels were not affected by increasing levels of dietary CSM. The presence of different levels of solvent extracted camelina meal in the diets did not cause any difference in fT3 and fT4 hormone levels and fT3/fT4 ratio in serum.

**Table 4:** Effects of high levels of solvent extracted CSM on some hematological parameters and some blood serum biochemical indices in broilers

Item	Solvent extracted CSM (%)					*p* value		
	0	10	15	20	25	Combined	Linear	Quadratic
Hematological parameters								
Hematocrit (%)	32.50 ± 0.93	32.33 ± 0.67	32.75 ± 0.78	32.25 ± 0.63	31.50 ± 0.74	0.820	0.389	0.466
Hemoglobin (g/dl)	10.13 ± 0.63	11.76 ± 0.46	11.89 ± 0.56	11.54 ± 0.66	11.40 ± 0.62	0.233	0.216	0.073
Heterophiles (%)	39.92 ± 2.64	40.08 ± 2.48	43.69 ± 2.07	44.09 ± 2.00	45.52 ± 2.06	0.304	0.038	0.928
Lymphocytes (%)	59.75 ± 2.61^a^	57.17 ± 2.06^ab^	51.58 ± 2.08^ab^	50.58 ± 2.24^b^	49.92 ± 2.28^b^	0.009	0.001	0.325
H/L	0.70 ± 0.07^b^	0.72 ± 0.05^b^	0.88 ± 0.07^ab^	0.89 ± 0.05^ab^	0.94 ± 0.06^a^	0.026	0.002	0.571
Eosinophiles (%)	4.18 ± 0.34	4.43 ± 0.39	4.59 ± 0.39	4.75 ± 0.49	5.53 ± 0.37	0.182	0.021	0.485
Basophiles (%)	1.58 ± 0.13	1.68 ± 0.13	1.73 ± 0.15	1.88 ± 0.13	1.91 ± 0.19	0.464	0.066	0.904
Monocytes (%)	4.17 ± 0.48	4.43 ± 0.52	4.43 ± 0.33	4.79 ± 0.34	4.80 ± 0.41	0.801	0.229	0.921
Blood serum biochemical indices								
TAS (mmol/L)	1.32 ± 0.06	1.41 ± 0.04	1.44 ± 0.04	1.43 ± 0.07	1.39 ± 0.06	0.587	0.372	0.163
TOS (µmol/L)	6.89 ± 0.52	6.95 ± 0.33	6.92 ± 0.44	6.93 ± 0.27	6.97 ± 0.43	0.999	0.912	0.994
OSI	0.53 ± 0.04	0.50 ± 0.03	0.49 ± 0.04	0.50 ± 0.03	0.51 ± 0.03	0.919	0.687	0.399
Total cholesterol (mg/dl)	87.50 ± 3.62^b^	94.57 ± 2.88^ab^	99.43 ± 2.38^a^	95.86 ± 2.33^ab^	101.14 ± 2.17^a^	0.008	0.002	0.244
Total protein (g/dl)	4.80 ± 0.06	4.86 ± 0.05	4.83 ± 0.05	4.79 ± 0.04	4.78 ± 0.04	0.656	0.448	0.334
Albumin (g/dl)	2.35 ± 0.03^b^	2.47 ± 0.04^a^	2.48 ± 0.02^a^	2.49 ± 0.03^a^	2.48 ± 0.03^a^	0.005	0.004	0.021
Triglyceride (mg/dl)	32.79 ± 2.77	39.86 ± 2.42	34.86 ± 2.13	41.29 ± 2.30	35.71 ± 2.90	0.107	0.079	0.092
IgA (mg/dl)	2.05 ± 0.25^b^	2.59 ± 0.18^ab^	2.78 ± 0.17^a^	2.79 ± 0.11^a^	2.81 ± 0.18^a^	0.020	0.004	0.082
IgM (mg/dl)	3.48 ± 0.15	3.78 ± 0.24	3.27 ± 0.26	3.08 ± 0.27	3.62 ± 0.23	0.246	0.166	0.117
IgG (µg/ml)	2.51 ± 0.19^b^	2.91 ± 0.26^ab^	3.08 ± 0.23^ab^	2.98 ± 0.29^ab^	3.88 ± 0.25^a^	0.005	0.001	0.235
fT3 (pg/ml)	11.60 ± 0.37	11.09 ± 0.33	11.55 ± 0.40	11.35 ± 0.49	11.57 ± 0.50	0.903	0.797	0.615
fT4 (pg/ml)	16.66 ± 0.78	17.00 ± 0.67	17.75 ± 0.73	17.58 ± 0.50	18.09 ± 0.56	0.544	0.102	0.816
fT3/fT4	0.70 ± 0.02	0.66 ± 0.01	0.66 ± 0.02	0.65 ± 0.02	0.64 ± 0.02	0.143	0.024	0.302

*n *= 12 for each group, values within a row with different superscripts (a, b) differ significantly at *p* < 0.05

*CSM Camelina sativa* meal, *H/L* the ratio of heterophiles to lymphocytes, *TAS* total antioxidant status, *TOS* total oxidant status, *OSI* oxidative stress index, *IgA* Immunoglobulin A, *IgM* Immunoglobulin M, *IgG* Immunoglobulin G, *fT3* free Triiodothyronine, *fT4* free Thyroxine

While no difference was observed in the counts of *E. coli* and *Lactobacillus* in the cecum, significant differences (*p* < 0.05) were observed between the groups in total bacteria and *Enterococcus* counts (Table 5). The use of solvent extracted CSM at 10, 15, 20, and 25% levels led to an increase in the number of *Enterococcus* in the cecum (*p* < 0.001). Linear and quadratic effects (P < 0.05) were also seen in the counts of total aerobic bacteria, *E. coli*, and *Enterococcus* in the cecum. As seen in Table 5, a significant linear increase (*p* = 0.001) was detected in the viscosity of ileum and jejunum digesta with the increasing levels of CSM in the diet.

**Table 5:** Effects of high levels of solvent extracted CSM on cecal microorganisms and intestinal viscosity in broilers

Item	Solvent extracted CSM (%)					*p* value		
	0	10	15	20	25	Combined	Linear	Quadratic
Cecal microorganisms (log_10_cfu/g)								
Total aerobic bacteria	6.87 ± 0.08^b^	7.19 ± 0.06^ab^	7.21 ± 0.10^a^	7.13 ± 0.11^ab^	6.90 ± 0.07^ab^	0.010	0.004	< 0.001
* E. coli*	4.18 ± 0.12	4.53 ± 0.10	4.58 ± 0.09	4.41 ± 0.12	4.23 ± 0.13	0.064	0.032	0.004
Enterococcus	4.06 ± 0.33^b^	5.67 ± 0.28^a^	5.38 ± 0.15^a^	5.59 ± 0.17^a^	5.32 ± 0.12^a^	< 0.001	< 0.001	< 0.001
* Lactobacillus*	4.87 ± 0.09	5.39 ± 0.13	5.33 ± 0.13	5.28 ± 0.17	5.20 ± 0.18	0.100	0.093	0.030
Viscosity (cP)								
Jejunum	0.70 ± 0.03^d^	1.13 ± 0.08^c^	1.72 ± 0.09^b^	1.85 ± 0.09^b^	2.42 ± 0.08^a^	< 0.001	< 0.001	0.052
Ileum	0.71 ± 0.05^e^	1.44 ± 0.09^d^	1.91 ± 0.09^c^	2.44 ± 0.09^b^	2.92 ± 0.09^a^	< 0.001	< 0.001	0.159

*n* = 12 for each group, values within a row with different superscripts (a, b, c, d, e) differ significantly at *p* < 0.05

*CSM Camelina sativa* meal

The use of solvent extracted camelina meal resulted in a reduction (*p* < 0.001) in villus height of the duodenum, jejunum, and ileum (Table 6). No change was observed in crypt depths of the duodenum, jejunum, and ileum. The villus height/crypt depth ratio decreased in the jejunum, and ileum with the use of solvent extracted camelina meal, however, no changes were observed in the duodenum among groups. Linear effects were seen in villus height and the ratio of VH/CD of the duodenum, jejunum, and ileum.

**Table 6:** Effects of high levels of solvent extracted CSM on intestinal histomorphology in broilers

Item	Solvent extracted CSM (%)					*p-*value		
	0	10	15	20	25	Combined	Linear	Quadratic
Duodenum								
Villus height (µm)	1601.74 ± 6.70^a^	1567.2 ± 5.98^b^	1568.76 ± 6.16^b^	1534.40 ± 9.59^c^	1531.18 ± 7.85^c^	< 0.001	< 0.001	0.097
Crypt depth (µm)	132.73 ± 1.81	136.06 ± 2.47	136.39 ± 2.86	133.06 ± 1.99	136.14 ± 2.91	0.701	0.589	0.429
VH/CD	12.08 ± 0.14	11.54 ± 0.23	11.53 ± 0.24	11.55 ± 0.21	11.28 ± 0.27	0.167	0.031	0.499
Jejunum								
Villus height (µm)	926.03 ± 9.37^a^	872.43 ± 9.29^b^	870.24 ± 9.69^b^	862.03 ± 8.80^b^	847.43 ± 7.52^b^	< 0.001	< 0.001	0.042
Crypt depth (µm)	152.86 ± 2.29	155.44 ± 2.22	155.33 ± 2.55	152.86 ± 2.29	149.27 ± 1.77	0.313	0.180	0.092
VH/CD	6.06 ± 0.08^a^	5.62 ± 0.10^b^	5.61 ± 0.10^b^	5.65 ± 0.10^b^	5.68 ± 0.06^b^	0.006	0.014	0.006
Ileum								
Villus height (µm)	607.86 ± 6.11^a^	571.19 ± 4.71^b^	570.39 ± 3.93^b^	553.53 ± 4.63^b^	554.02 ± 5.82^b^	< 0.001	< 0.001	0.005
Crypt depth (µm)	140.31 ± 1.78	140.80 ± 1.05	142.12 ± 1.08	140.21 ± 1.33	138.73 ± 1.48	0.543	0.397	0.175
VH/CD	4.33 ± 0.03^a^	4.06 ± 0.04^b^	4.02 ± 0.04^b^	3.95 ± 0.06^b^	4.00 ± 0.04^b^	< 0.001	< 0.001	0.001

*n* = 6 for each group, values within a row with different superscripts (a, b, c) differ significantly at *p* < 0.05

*CSM Camelina sativa* meal, *VH/CD* villus height /crypt depth

It is seen in Table 7 that increasing the dietary camelina meal linearly decreased (*p* < 0.001) the DM, CP, and gross energy digestibility in each of the periods in the experiment.

**Table 7:** Effects of high levels of solvent extracted CSM on diet digestibility in different periods in broilers

Item	Solvent extracted CSM (%)					*p-*value		
	0	10	15	20	25	Combined	Linear	Quadratic
0–10 day								
DMD (%)	71.58 ± 0.74^a^	65.28 ± 0.80^b^	59.38 ± 0.80^c^	58.90 ± 0.74^c^	56.84 ± 0.79^c^	< 0.001	< 0.001	< 0.001
CPD (%)	70.23 ± 0.63^a^	66.55 ± 0.90^a^	59.14 ± 0.99^b^	58.63 ± 0.94^b^	56.64 ± 0.98^b^	< 0.001	< 0.001	0.005
GED (%)	71.97 ± 0.86^a^	65.72 ± 0.80^b^	61.59 ± 0.99^c^	60.85 ± 0.89^cd^	57.86 ± 0.58^d^	< 0.001	< 0.001	0.004
10–24 day								
DMD (%)	69.39 ± 0.69^a^	65.55 ± 0.79^b^	62.16 ± 0.85^c^	58.77 ± 0.77^d^	58.28 ± 0.66^d^	< 0.001	< 0.001	0.025
CPD (%)	71.09 ± 0.95^a^	68.68 ± 0.72^ab^	65.52 ± 0.81^bc^	63.32 ± 0.89^c^	59.69 ± 0.90^d^	< 0.001	< 0.001	0.649
GED (%)	71.97 ± 0.62^a^	67.11 ± 0.86^b^	64.04 ± 0.87^bc^	62.06 ± 0.99^c^	60.96 ± 0.55^c^	< 0.001	< 0.001	0.007
24–42 day								
DMD (%)	70.95 ± 0.54^a^	66.78 ± 0.87^b^	64.45 ± 0.91^bc^	63.03 ± 0.81^c^	56.40 ± 0.74^d^	< 0.001	< 0.001	0.022
CPD (%)	65.91 ± 1.36^a^	63.07 ± 1.00^ab^	58.51 ± 1.50^bc^	57.34 ± 1.39^c^	51.71 ± 1.18^d^	< 0.001	< 0.001	0.402
GED (%)	75.82 ± 0.38^a^	71.62 ± 0.78^b^	69.91 ± 0.78^b^	69.86 ± 0.67^b^	63.95 ± 0.44^c^	< 0.001	< 0.001	0.001

*n* = 6 for each group, values within a row with different superscripts (a, b, c, d) differ significantly at *p* < 0.05

*CSM Camelina sativa* meal, *DM* dry matter, *DMD* dry matter digestibility, *CPD* crude protein digestibility, *GED* gross energy digestibility

## Discussion

*Camelina sativa* meal used in the present study was obtained after oil extraction for biodiesel production; therefore its oil content is low. GSLs form in the meal is important for the toxicity. Long-chain GSLs are predominant in *Camelina sativa* products, and they do not induce toxic effects as those found in rapeseed (Matthaus and Zubr 2000; Oryschak et al. 2020). The level of total GSLs in the Arslanbey cultivar of camelina meal was found to be lower than the findings of some researchers determined in camelina and camelina products (Woyengo et al. 2016; Oryschak et al. 2020), higher than Ryhanen et al. (2007) and seen within the range of findings that stated by Russo and Reggiani (2017) and Schuster and Friedt (1998). Russo and Reggiani (2017) reported that sinapine ranged from 1.09 to 4.75 g/kg dry weight of forty-seven samples of defatted camelina (*Camelina sativa* (L.) Crantz) and concluded that sinapine content was not related to GSL levels and that the use of camelina meal is primarily restricted by GSL level, with sinapine content being less of a concern in camelina varieties. For the biosynthesis of GSLs, sulphur-containing amino acids are required; therefore, soil rich in sulphur led to increased GSLs content of camelina seeds (Schuster and Friedt 1998).

Differences in nutrient composition are observed between the CSM used in this study and *Camelina sativa* used in other studies (Ryhanen et al. 2007; Bulbul et al. 2015; Pekel et al. 2015; Woyengo et al. 2016; Oryschak et al. 2020). In these studies, *Camelina sativa* meal or cake had high oil content (6.44–36.84%). The crude fibre level (10.80%) of camelina meal in the present study was in the range of 9.7–17.40% fibre that was reported in the studies (Ryhanen et al. 2007; Pekel et al. 2015; Bulbul et al. 2015; Woyengo et al. 2016; Oryschak et al. 2020). These differences might also be attributed to seed genotypes, climate, soil, and the soil fertilization (Schuster and Friedt 1998; Matthaus and Zubr 2000).

Linear reduction (*p* < 0.001) in live weight, weight gain, and feed consumption was observed during the experiment. Chicks fed with 25% CSM consumed more feed per each kg live weight gain than the other groups during the first 10-day period. After the first period, when the first 24 days and the total 42 days of the trial were evaluated, it was seen that feed efficiency was better in the control group than in the other groups (*p* < 0.001) and that feed efficiency decreased linearly with the increasing doses of CSM level (*p* < 0.001). These results are in agreement with Pekel et al. (2015), who reported a reduction in the criteria of growth performance. Similarly, 10% *Camelina sativa* expeller (Ryhanen et al. 2007) and 3–15% camelina cake (Thacker and Widyaratne 2012) reduced feed intake and growth in broilers. However, no effect of dietary 10% of camelina meal on performance was observed by Aziza et al. (2010). The poorer performance may be due to the GSLs content of *Camelina sativa* (Ryhanen et al. 2007; Thacker and Widyaratne 2012; Pekel et al. 2015). Feeds containing high levels of GSLs have been shown to reduce feed intake and growth rate (Verhoeven et al. 1997). GSLs are relatively unpalatable and they and their breakdown products determine the typical flavour and bitter taste of the Brassica species (EFSA 2008).

EFSA (2008) reported that broiler growth decreased over the 37-day experiment with increasing levels of *Camelina sativa* cake (0, 5, 10, 15, 20, and 25%), with the most pronounced reduction observed in diets containing 20% and 25% camelina cake. Feed intake differences among groups fed 0, 5, 10, 15, and 20% cake were relatively minor, while a notable decrease in feed intake was evident in broilers receiving 25% camelina cake. The differences among studies are due to differences in the processing of *Camelina sativa* seeds. The processing influences the contents and mode of action of antinutritional compounds and the content of lipids and fatty acids.

Inclusion of CSM up to 25% did not affect liveability during the 42 day experiment. However, one death in each group containing 15% and 25% camelina meal during the experiment was observed but the deaths were not related to any dietary treatment. Similarly, some researchers demonstrated that mortality was not affected by 10% (Pekel et al. 2009) and higher levels up to 24% (Oryschak et al. 2020) camelina cake in broiler diets. This indicates that the antinutritional compounds contained in *Camelina sativa* are not high enough to cause death. The levels of EPEF, which is a performance indicator, of the groups fed CSM were lower (*p* < 0.001) than that of the control group, and linear reduction was also seen with the increasing levels of CSM in the diets. However, low inclusion level (5%) did not cause negative effects (Valkonen et al. 2004).

In the present study, carcass yield was reduced, and the relative weight percentages of the heart, proventriculus, gizzard, and thyroid gland were increased linearly with increasing levels of CSM in the diet. In contrast to the study, carcass yield was not affected significantly by the usage of 10% camelina meal in broilers (Pekel et al. 2009). Similar to the present study, Oryschak et al. (2020) reported that relative weight values of liver and spleen were not affected, but relative weight values of the heart linearly increased with increasing dietary inclusion of *Camelina sativa*. The increased gizzard weight seen in the current study could be related to the dietary fiber content associated with *Camelina sativa* (Gebeş et al. 2024).

In contrast to the present study, some researchers (EFSA 2008; Ciurescu et al. 2016) showed that the abdominal fat proportion decreased linearly with increasing dietary *Camelina sativa* cake. Ciurescu et al. (2016) suggested that a diet containing *Camelina sativa* rich in oil (high in PUFA) causes to reduce abdominal fat deposition.

A high content of dietary GSLs may affect thyroid functions (Ryhanen et al. 2007). Similar to the present study, in the report of EFSA (2008) the relative weights of the thyroid glands were shown to increase linearly with increasing *Camelina sativa* cake content in feed (20–25%).

The haematologic and serum biochemical results are usually considered to assess the health status of an animal. Thiam et al. (2021) demonstrated that the H/L ratio correlates with intestinal barrier function and immune response, with broilers exhibiting a lower H/L ratio showing improved intestinal immunity. The increase in H/L ratio with the increase in *Camelina sativa* dose in the present study may be due to the antinutritional substances, especially GSLs.

Similarly, the reduction in total cholesterol also obtained by Anca et al. (2019) in broilers for 8% camelina cake. In contrast to the current study, Orczewska-Dudek et al. (2019) showed a tendecy towards a reduction of the plasma content of cholesterol and triglycerides in the broilers fed a diet containing 10% *Camelina sativa* cake but these reductions were not found to be significant.

In contrast to the present study, Bulbul et al. (2015) reported that 5, 10, 15, and 20% camelina meal inclusion in the diets of quails reduced serum antioxidant activity levels linearly attributed to the presence of antioxidant compounds and other bioactive compounds such as tocopherols and phenolics. Antinutritional factors, especially GSLs in *Camelina sativa* meal, may have hindered the release of antioxidant compounds in the body and thereby prevented an improvement in the bird’s antioxidant status in the present study. The differences may also be due to the variety of *Camelina sativa*, the production procedure of the meal, and the levels of oil content and fatty acid profile, and fiber content.

In the present study, serum IgG and IgA levels were increased by CSM inclusion. This situation may be useful in commercial broiler production. However, in the study of Ciurescu et al. (2016) plasma IgG, IgM, and IgA levels were not affected with the use of 5% and 10% camelina seed in the diets of broilers.

Thyroid hormones play an important role in controlling basal metabolism and for proper growth and development (Darras et al. 2000). The less bioactive thyroxine (T4) form is generally present in serum in greater concentrations than the more bioactive triiodothyronine (T3) form (Moravej et al. 2006; Oryschak et al. 2020). In contrast to the present study, Oryschak et al. (2020) found that serum levels of T3 decreased and T4 increased with increasing doses of *Camelina sativa*. However, in the study of Oryschak et al. (2020), *Camelina sativa* was added to the diets as top-dressed and therefore CP content of diets increases with the increasing levels of *Camelina sativa* and the differences in T3 and T4 levels may be due to the difference in CP and digestible nutrient levels in the diets. Orczewska-Dudek et al. (2019) showed that 10% camelina sativa increased plasma T3 and T4 levels, and significant increases (*p* < 0.05) were seen in plasma T3 levels. GSLs block thyroid hormone receptors and induce changes in the outer ring deiodination of T4 in peripheral tissues (Darras et al. 2000). Then the concentration of T3 decreases. According to the study of Moriel et al. (2011), different levels of camelina cake did not affect hormone levels in the blood of cows. In contrast, Lardy and Kerley (1994) reported that GSLs cause the reduction in the circulating T3 and T4 levels.

The cecum plays a crucial role in functional activity having its slower intestinal flow compared to other segments and having a high capacity for water absorption and carbohydrate fermentation. These properties enhance the taxonomic diversity of microbiota, that is vital for health and performance (Rubio et al. 2015). However, under certain conditions, undesirable microbiota including *Enterobacteriaceae* such as *E. coli* and *Salmonella*, can also proliferate in the cecum (Mon et al. 2015; Zajac et al. 2021). According to Zajac et al. (2021), *E. coli* was not detected in the cecum and cloaca of broilers supplemented with micronized camelina seeds. They suggested that the restricted growth and diversity of *Enterobacteriaceae* in these areas might reflect the sensitivity of other microbial taxa to the antimicrobial properties of compounds found in camelina seeds. Additionally, Zajac et al. (2021) noted that substances with known antibacterial effects, such as polyunsaturated fatty acids, are present in camelina seeds. Mazanko et al. (2023) also demonstrated that *Camelina sativa* oil cake has prebiotic effects on the broiler microbiome in model studies. It boosts the acid production of lactic acid bacteria, which lowers the pH of the medium. This results in a reduction of counts of *Enterococcus*, *E. coli*, and lactose-positive bacteria and completely suppresses Proteus (Mazanko et al. 2023). Mazanko et al. (2023) concluded that oil cake of *Camelina sativa* is a promising source of prebiotics for the development of prebiotic feed. This situation was not seen in the present experiment that solvent extracted camelina meal having low oil content. In the present experiment linear increases were seen in total aerobic bacteria, *E. coli*, and *Enterococcus* in the cecum with increasing doses of CSM and no significant changes were seen in *Lactobacillus* count.

Similar to the present study, Pekel et al. (2015) found that adding 10% and 20% camelina meal to the diet increased jejunal digesta viscosity. This higher viscosity may have negatively impacted growth (Pekel et al. 2015) like in the current study. According to the study of Bedford and Classen (1993), viscosity is linked to the content of nonstarch polysaccharides in the diet. Adeola and Bedford (2004) demonstrated that reduced growth performance is associated with increased intestinal digesta viscosity, which leads to decreased energy utilization and absorption of nutrients. Some researchers (Rodriguez et al. 2001; Alzueta et al. 2003) indicated that lower digestibility was due to the high levels of water soluble polysaccharides such as mucilage, which increased the viscosity of intestinal contents.

Small intestine segments are important parts for nutrient digestion and absorption. To maximize the effectiveness of feeding strategy in enhancing gut health, nutrient utilization, and thereby performance, morphological characteristics of the small intestine segments in broilers must be considered. The length of intestinal villi and crypt depth are indicative of the nutrient digestion and absorptive capacity of the intestine (Aziza et al. 2014; Yalçın et al. 2022). The increase in villus height is paralleled by an increase in digestive and absorptive functions and bird growth. Short villi are correlated with reduced nutrient digestion and absorptive capacity and thereby performance was reduced. The VH/CD ratio is considered to be a useful criterion for estimating the digestive capacity of the small intestine and a low VH/CD ratio suggests a poorly differentiated intestinal mucosa with low digestive and absorptive capability (Aziza et al. 2014; Yalçın et al. 2022). The observation of low VH/CD and high CD also confirms that blood H/L ratio is high with the increase in the dose of *Camelina sativa*. In this context, the low VH/CD ratio in CSM groups causes poorer growth as seen in the present study. Antinutritional factors such as GSLs from CSM could hinder bird’s ability to absorb nutrients, affecting its growth performance (Aziza et al. 2014). As shown in the present study, Aziza et al. (2014) reported lower villus height and the ratio of VH/CD and higher crypt depth in broilers fed 10% camelina meal than those of the control group.

Similar to the present study, Oryschak et al. (2020) reported that apparent total tract digestibility values of gross energy, DM and CP were decreased linearly by the increasing levels (0, 8, 16, and 24%) of screw-pressed *Camelina sativa*. Aziza et al (2013) found that total tract CP digestibility of a diet having 10% camelina cake (0.28) was lower than the diet having corn-soybean meal (0.40) in laying hens. Thacker and Widyaratne (2012) also demonstrated that 3% camelina cake inclusion did not affect the nutrient digestibility; however, there was a linear reduction in apparent total tract digestibility of DM, gross energy and nitrogen retention in broilers fed increasing dietary levels of camelina cake from 3 to 15%. Acamovic et al. (1999) similarly found low digestibility coefficients for DM and nitrogen in camelina cake for broilers.

The reduction in energy and nutrient digestibility may be attributed to the increased fiber content in the diet. High dietary fiber can impair digestibility due to its physicochemical properties, leading to a faster passage rate that limits nutrient absorption time (Burkett et al. 1972). Similarly, Pekel et al. (2015) reported that increasing levels (10, 20%) of camelina meal inclusion in the diets linearly decreased the ileal digestibility of DM, energy and nitrogen and also metabolizability of DM, nitrogen and energy. Thacker and Widyaratne (2012) reported that increasing levels (3, 6, 9, 12 and 15%) of camelina meal inclusion in the diets linearly decreased the apparent total tract digestibility of DM, energy, and nitrogen. Aziza et al. (2013) reported decreased digestibility of DM and CP. Singh et al. (2023) reviewed that the reduction in nutrient digestibility could be attributable to the presence of non-starch polysaccharides in the camelina cake, responsible for increasing the digesta viscosity. Inconsistent research findings may be attributed to the variations in rearing and climatic conditions of *Camelina sativa*, cake/meal production methods, and doses of camelina in experiments.

## Conclusion

The present study reveals that dietary usage of solvent extracted CSM obtained after biodiesel production significantly reduced the growth and feed efficiency compared with those fed soya. These negative effects may be through reductions in feed intake and nutrient digestibility. A higher level of GSLs and fibre in CSM compared with soyabean meal/fullfat soya may explain these findings. By removing negative effects of antinutritional compounds in CSM, camelina can become an alternative protein source for broilers. Further research is required to fully understand the optimal inclusion level and proper processing methods for camelina meal in poultry diets to improve sustainability in broiler production.

## Data Availability

The data that support the findings of this study are available from the corresponding author upon reasonable request.
